# Supplementary material for: Defective membrane repair machinery impairs survival of invasive cancer cells
Source: Sci Rep. 2020 Dec 11;10:21821. doi: 10.1038/s41598-020-77902-5 (PMC7733495; doi:10.1038/s41598-020-77902-5)
Supplement: Supplementary file 1 — Supplementary Information 1. [file 41598_2020_77902_MOESM1_ESM.docx]

**Electronic supplementary material**

**Supplementary Fig. 1** presents the cell culture device used for membrane repair assay. **Supplementary Fig. 2** presents the expression of endogenous Anx in MDA-MB-231 cells analyzed by western-blot. **Supplementary Fig. 3** shows that MDA-MB-231 cells that express AnxA5 at a high level express also strongly AnxA6 and, inversely, a low level of AnxA5 is systematically accompanied by a weak expression of AnxA6. **Supplementary Fig. 4** shows that the decrease of the expression of AnxA5 or AnxA6 in knocked-down MDA-MB-231 cells is about 90%. **Supplementary Fig. 5** presents western-blot experiment showing that MDA-MB-231 cells transduced together with AnxA5- and AnxA6- targeting shRNAs exhibit a decrease by about 73% and 99% for AnxA5 and AnxA6, respectively. **Supplementary Fig. 6** shows the response of AnxA5-AnxA6 deficient MDA-MB-231 cells to a membrane damage by laser ablation, which leads to conclude that these cells suffer from a defect of membrane repair. **Supplementary videos 1 and 2** present the migration of MDA-MB-231 cells respectively in the presence and in the absence of collagen I using phase-contrast video-microscopy. **Supplementary video 3** presents a cell that have migrated on fibrillar collagen I, for which CellMask staining revealed the presence of membrane fragments in its wake. **Supplementary video 4** shows the important variations of fluorescence intensity in Fluo-4-AM loaded MDA-MB-231 cells migrating on collagen I using fluorescence video-microscopy. **Supplementary video 5** shows the responses of MDA-MB-231 cells to a membrane damage by laser ablation. **Supplementary video 6** shows that AnxA5-AnxA6 deficient MDA-MB-231 cells become rounded as soon as they are starting to migrate in collagen I, using phase-contrast video-microscopy. **Supplementary video 7** shows the behavior of AnxA5-AnxA6 deficient MDA-MB-231 cells on gelatin, ie in the absence of fibrillar collagen I. **Supplementary video 8** shows by fluorescence video-microscopy that Fluo-4-AM loaded AnxA5-AnxA6 deficient MDA-MB-231 cells exhibits repeated variations of the intracellular fluorescence.
